# Standardization meets stories: Contrasting perspectives on the needs of frail individuals at a rehabilitation unit

**DOI:** 10.3402/qhw.v8i0.21498

**Published:** 2013-09-20

**Authors:** Bente Prytz Mjølstad, Anna Luise Kirkengen, Linn Getz, Irene Hetlevik

**Affiliations:** 1 General Practice Research Unit, Department of Public Health and General Practice, Norwegian University of Science and Technology (NTNU), Trondheim, Norway; 2 Institute of Community Medicine, University of Tromsø, Tromsø, Norway; 3 Centre for Health Promotion, Akershus University Hospital, Nordbyhagen, Norway

**Keywords:** Biographical knowledge, lifetime experience, phenomenology, general practice, rehabilitation, standard treatment, person-centered medicine, narrative medicine

## Abstract

**Background:**

Repeated encounters over time enable general practitioners (GPs) to accumulate biomedical and biographical knowledge about their patients. A growing body of evidence documenting the medical relevance of lifetime experiences indicates that health personnel ought to appraise this type of knowledge and consider how to incorporate it into their treatment of patients. In order to explore the interdisciplinary communication of such knowledge within Norwegian health care, we conducted a research project at the interface between general practice and a nursing home.

**Methods:**

In the present study, nine Norwegian GPs were each interviewed about one of their patients who had recently been admitted to a nursing home for short-term rehabilitation. A successive interview conducted with each of these patients aimed at both validating the GP's information and exploring the patient's life story. The GP's treatment opinions and the patient's biographical information and treatment preferences were condensed into a biographical record presented to the nursing home staff. The transcripts of the interviews and the institutional treatment measures were compared and analysed, applying a phenomenological–hermeneutical framework. In the present article, we compare and discuss: (1) the GPs’ specific recommendations for their patients; (2) the patients’ own wishes and perceived needs; and (3) if and how this information was integrated into the institution's interventions and priorities.

**Results:**

Each GP made rehabilitation recommendations, which included statements regarding both the patient's personality and life circumstances. The nursing home staff individualized their selection of therapeutic interventions based on defined standardized treatment approaches, without personalizing them.

**Conclusion:**

We found that the institutional voice of medicine consistently tends to override the voice of the patient's lifeworld. Thus, despite the institution's best intentions, their efforts to provide appropriate rehabilitation seem to have been jeopardized to some extent.

Modern medicine is grounded in the natural sciences’ understanding of human beings, from Newton and Descartes, through the 17th century Scientific Revolution, the Age of Enlightenment, 19th century physics and into 20th century molecular biology (Lock & Gordon, 1988). Within this perspective, body and mind are regarded as separate, and the person's life history and subjective experience are granted at most a “supplementary” status. To assure quality and cost control, diagnosis and treatment are increasingly determined and evaluated using a set of standards rooted in statistical knowledge about groups, rather than in explorations of the needs of individual patients. This “depersonalized” approach has indisputably contributed to breakthroughs and a well-proven practical efficacy in the treatment of many well-defined medical problems. As an approach to human health and disease generally, however, it may not be adequately comprehensive and may lack validity (Cassell, 2004; Kirkengen & Thornquist, 2012; Zaner, 2003a). This depersonalized and group-based knowledge shows, in fact, its crucial limitations as we are currently witnessing the rapid growth of scientific evidence documenting both that, and how, an individual's lifetime experiences and existential circumstances have a significant impact on health (Felitti & Anda, 2010; Glaser & Kiecolt-Glaser, 2005; Gruenewald et al., 2012; McEwen & Getz, 2013; Miller, Chen, & Parker, 2011; Norman et al., 2012; Seeman, Epel, Gruenewald, Karlamangla, & McEwen, 2010; Shonkoff, Boyce, & McEwen, 2009; Steptoe & Marmot, 2002; Surtees et al., 2011). Knowledge about the fundamental and reciprocal interrelatedness of human biology and biography (Getz, Kirkengen, & Ulvestad, 2011) may be of particular relevance to the treatment of patients suffering from ill-defined and/or complex health problems (Eriksen, Kirkengen, & Vetlesen, 2013; Kirkengen, 2001). It may also have implications, which are crucial to the care of frail human beings who have decompensated (in terms of functional impairment) to such an extent that rehabilitative institutional care is required. The present study focuses on such a situation.

The field of general practice/family medicine, wherein continuity of care is built upon repeated personal encounters, may well be where the incongruity between the dominant biomedical paradigm (as described above), and the real-life challenges of everyday medical practice becomes most overt. Encountering patients over the course of years, general practitioners (GPs) are likely to gain biographical knowledge with major relevance for the patient's life and health, whether learned coincidentally and perhaps not even recognized as important, or elicited intentionally based on a genuine insight into its potential relevance (Kirkengen, 2008). Over decades, several pioneers in general practice/family medicine have argued for more comprehensive medical models and approaches which could integrate knowledge regarding the patients’ context and lifeworld. The most well-known of these are the “bio-psycho-social model” (Engel, 1977) and “patient-centered medicine” (Levenstein, McCracken, McWhinney, Stewart, & Brown, 1986). More recently, the emphasis has begun to shift from the *patient* to the *person*, as reflected in the new terms “person-centered” (Miles & Mezzich, 2011) and “person-focused” medicine (Starfield, 2011). It has been postulated that this emerging interest in the needs of the particular individual, as opposed to an “average” patient, has come in reaction to an on-going dehumanization of medicine as an increasingly predominating focus on standardized technological *cure* may be in danger of taking precedence over attention to individualized human *care* (Kirkengen, Mjølstad, Getz, Ulvestad, & Hetlevik, 2013; Miles & Mezzich, 2011).

## Medical rehabilitation of frail individuals—cure or care?

The difference between a standardized “cure” and a person-centered “care” approach might be explored fruitfully in the context of institutional health care settings, focusing on individuals who are experiencing deterioration in health and function. This group includes individuals who have become frail prematurely due to chronic debilitating conditions, typically more than one (Barnett et al., 2012). The frailty of others in this group may be due to their advanced age or the combination of age and multi-morbidity (Martin et al., 2012; Sturmberg, 2012). Today's elderly are generally in better health and function at a higher level for longer than did previous generations. Nevertheless, as the aging population increases, more elderly and frail people are likely to find themselves “in transit” between home and institutions. These patients both want and need to be met by professionals who can coordinate an individualized care plan which takes the specific patient's needs into account (Bayliss, Edwards, Steiner, & Main, 2008). Consequently, an exploration of what kind of knowledge is considered relevant for the patient's GP to transmit to the caretaking institution, when a fragile individual is admitted, is both timely and useful, from a scientific as well as a practical point of view.

## Context for the present study

In Norway, where this study was conducted, strong emphasis is currently placed on providing home-based care to elderly and frail people. Within a formal health care perspective, and with governmental support, rehabilitation is conceptualized as: planned, time-limited processes in which several agents provide essential assistance, applying well-defined means to reach clearly delineated goals, supplementing the user's own efforts toward attaining the highest possible level of functioning and coping in terms of autonomy and of participation in a social life and in society (our translation) (Garåsen, 2008). Most frail or elderly people in Norway remain at home until they reach a critically low level of cognitive and/or physiological functioning, at which point the likelihood of being admitted to an institution increases substantially. This is largely congruent with the findings of Gaugler and colleagues (2007) suggesting a threshold model that may predict nursing home admission.

The most appropriate institutions to receive frail patients at such junctures are the so-called nursing homes, some of which have specialized “rehabilitation units.” In both settings, time-limited care is provided by an interdisciplinary staff. There exist no national guidelines for rehabilitation in nursing homes. However, in accordance with the definition and the understanding of the concept “rehabilitation,” the stated intention of these institutions is to offer individualized care based on comprehensive assessments resulting in a structured, individualized plan which includes therapeutic treatment designed to facilitate recovery. Usually, desirable outcomes (clear goals) are formulated and included in such plans. Specialized rehabilitation units evaluate each patient's condition systematically. Interdisciplinary collaboration, occupational therapists, physiotherapists and consulting physicians focus primarily on monitoring and improving the patients’ capacity to manage daily life activities (ADL). Most Norwegians are assigned to a specific GP (list system), a system which, ideally, assures continuity of care. When the patient is transferred from her/his home to a nursing home/rehabilitation unit, the institution formally requests the assigned GP to provide essential medical information including diagnoses, current medication, etc. Currently, no formalized standards regulate what type of biographical and contextual information should ideally follow patients to (or from) health care institutions. After admission to the nursing home/rehabilitation unit, the patient's treatment is turned over to the consulting physician (a GP or, rather infrequently, a specialist in rehabilitation medicine or geriatrics), who is connected to the institution.

## Aim of the present study

As the third step in a three-phased project (Mjølstad, Kirkengen, Getz, & Hetlevik, 2013a, b), the present study aims to explore the medical relevance of person-related knowledge both in general practice and at the interface between primary care and institutional care. In the initial phase, two groups of GPs were invited to reflect upon and discuss the potential significance of knowing their patients as persons. The GPs expressed confidence that they did possess medically relevant knowledge about their patients’ lifeworld, and that this knowledge might well have relevance for the health of patients admitted for rehabilitation (Mjølstad et al., 2013a). In the second phase, we explored what knowledge GPs *actually* had, by comparing the information provided by GPs to the narratives offered by the patients themselves (Mjølstad, Kirkengen, Getz, & Hetlevik, 2013b). In the present study, we compare and discuss three perspectives on the patients’ needs and aims when admitted to a rehabilitation unit, as described above: (1) what GPs recommended on behalf of some particular patient; (2) what those patients themselves considered central to their own functional improvement; and (3) how the institution responded to these individualized priorities.

## Theoretical framework

Researchers aiming at exploring and reflecting upon human experience in the context of medicine and medical practice would be well-advised to choose phenomenology as their theoretical framework (Kvale, 1983; Mishler, 1986). As a methodology, phenomenology allows for insight into the interviewee's world of personal experiences while at the same time maintaining and attending to the context. Experiences are always, a priori, experiences of something for somebody situated in a particular context. Consequently, the issue of personhood must be a central component in any research on human experience. While “patient” is a (reductive) role imposed on a person by disease and conceptualized in accordance with pathology-oriented biomedical theory, “personhood” as a status is constituted by other phenomena and rules. In our differentiation between “patient” and “person,” we apply Eric Cassell's (2010) view of a person as an “embodied, purposeful, thinking, feeling, emotional, reflective, relational, human individual always in action, responsive to meaning and whose life in all spheres points both outward and inward,” so that a person's behavior, whether “volitional, habitual, instinctual or automatic,” has its genesis from and in *meaning*. Since “meaning” and “personhood” are mutually constituting, statements about persons are statements about values and social phenomena. Any investigation of experience as communicated through first-person accounts involves encountering and exploring systems of values and of symbols as they are conceptualized and expressed in language, spoken, and written. Consequently, they demand a competence in hermeneutics (interpretations) (Kvale, 1983; Mishler, 1986, 1999).

Experience relates as much to the body as it is bound to the person; bodily being is the basic premise for experience, which is first perceived bodily and then interpreted personally. French philosopher and phenomenologist Maurice Merleau-Ponty (1989) regards the body, including when it is diseased and incapacitated, as embodied life—a lived body. This contrasts to the biomedical body, which is conceptualized as devoid of history and experience (Cassell, 1992). From a phenomenological perspective, rehabilitation might thus be understood as a personal, relational as well as bodily process, as the person's embodied, lived experiences. When searching for appropriate measures relating to a specific person, that person's lifeworld of subjective phenomena and inter-subjectively constituted values and symbols must inevitably be included among the premises (Zaner, 2003a). In the true sense of the word, “rehabilitation” signifies the means for “restoring a patient to the status of person” and “reinstating that person within the realm of dignity” (our translation) (Helse og Omsorgsdepartementet, 1997).

This project is distinctive not only by involving the interface between differing aspects of the health care system. It also takes place at the intersection between *cure* and *care*. The basic definition of rehabilitation alludes more to providing active medical treatment/therapy than to accommodating to people, or nursing them. *Curing*, in the sense of “treatment,” is the hegemonic realm of physicians while *caring* is the traditional province of nurses and other caretakers. This implies that the models and principles of biomedical knowledge production are the frame of reference for all interventions and treatment measures despite an apparent integration of cure and care in modern medicine. Still, between these domains, that of *cure* and that of *care*, there exists a demarcation line and an asymmetry of rank and authority.

## Methods and material

### Research site

This study was conducted in a rehabilitation unit at an urban nursing home in Mid-Norway with 32 single rooms for patients undergoing short-term rehabilitation (2–3 weeks). The staff included consulting physicians, nurses, physiotherapists, occupational therapists and nurses’ aides. The service provided was based on an interdisciplinary approach involving multiprofessional cooperation, with shared protocols but separated record keeping. In principle, records were data-based, but the various professional groups used different software systems as well as paper records. Information about the patient considered essential for the rehabilitation purpose was made accessible for all the professional groups. The patient her/himself (or family members) had to apply to be admitted (self-referral) with the Health and Welfare Agency in the city being responsible for granting permission. Accessible health information from the patient's GP and the community home care services was obtained and evaluated. If a patient had been hospitalized recently, the discharge letter was obtained.

An entry procedure was carried out, typically a dialogue with a nurse, aimed at identifying the patient's needs. The “mapping tool” included a checklist for the “patient care plan” as well as a questionnaire. The checklist contained a schedule, indicating the sequence of treatment measures and the distribution of tasks among staff members. The questionnaire addressed the following topics: actual health problems, mobility, ADL, family relations, social behavior/functioning, housing conditions, and the patient's own expectations and goals for rehabilitation. The nurse was mandated to delineate appropriate aims for the stay, resulting in a description of a primary goal. The primary goal was then broken down into several secondary goals. Finally, an individual rehabilitation plan, designed to take into account all of the collected information, was drawn up.

### Research design, data collection, and ethical approval

Only patients who were living at home when admitted for a rehabilitation stay were considered for inclusion. If the staff deemed a patient capable of giving informed consent, she/he was invited by the staff based on a preformulated invitation. Once the patient's consent was received, the researcher introduced herself to the patient, asked for permission to contact her or his regular GP for further information, and, provided permission, phoned the doctor for consent to discuss her/his knowledge regarding that patient as a person. Further information about the study was telefaxed to each GP's office along with a copy of the patient's signed consent form. After consent was provided, a 10–15-min telephone interview with the GP was scheduled within 3 days. This interview, based on two main questions, explored the GPs’ reflections concerning the most salient needs of this particular patient with regard to her/his rehabilitation (Mjølstad et al., 2013b). Each patient interview, performed face-to-face, took place shortly after the respective GP interview and lasted for approximately 1 hour. The departure point for each of these interviews was a condensed version of the information, which the GP had agreed the first author could share with the patient. The patient was encouraged to correct and/or deepen this information. In addition, the GP's proposal as to the central aim of the rehabilitation process was discussed with the patient. Based on these two integrated sources, the first author wrote a paper-based, biographical patient record, including a description of the patient as a person, the advice of the GP, and the explicit wishes of the patient regarding her/his rehabilitation. This record was then handed over to the staff member(s) responsible for the care of this patient, typically one of the consulting physicians and/or a nurse. The staff members were encouraged to consider this information in terms of appraising the biographical records when establishing the patient's rehabilitation plan. The patients and the health personnel had granted the first author access to the complete medical records of the participants.

**Figure 1:**
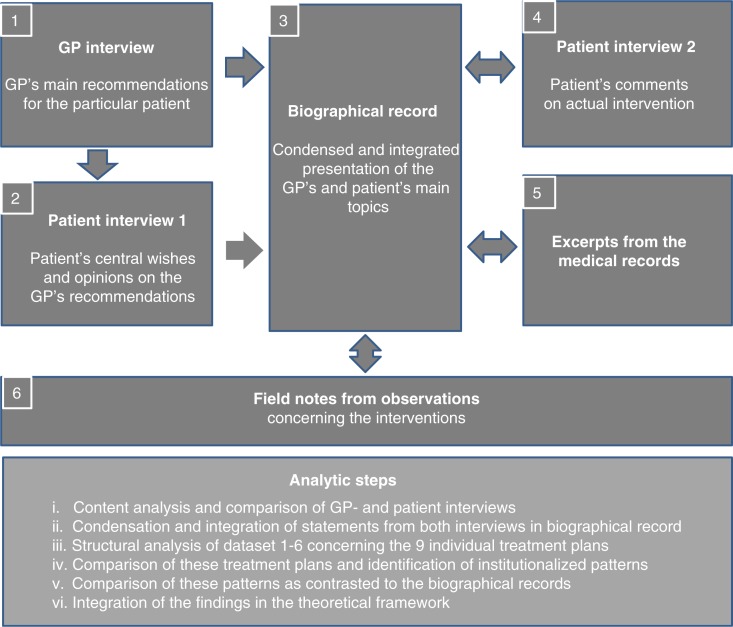
The components of data material (1–6) and description of the analytic steps (i–vi).

The first author recorded detailed and comprehensive notes regarding each of the included patients from the moment these had consented to participate and through her frequent visits during the entire period of data collection. The notes included reports after having talked with staff members and participated in unit staff-meetings concerning these patients. The notes also comprised observations, comments and reflections linked to the interview settings and to interactions with staff members. Finally, they were completed with excerpts from the patients’ electronic and paper-based records (including staff members’ notes). The first author was not given access to information about other patients than those included, or about other aspects of the unit, nor was she a regular observer of everyday routines or procedures. Her interest was not directed towards observing organizational or structural aspects or interaction among staff. An audio-taped and transcribed second interview with every patient regarding her/his final appraisal of the rehabilitation period completed the datasets for each of the nine persons included in the study. Thus, the complete materials consisted of: GP interviews, patient interviews (1 and 2), biographical records, excerpts from the medical records, and field notes (Figure 1).

The Regional Committee for Medical Research Ethics for Central Norway approved of the study, the collection of patient information, and the consent form structures (approval date 07.05.09). Prior to inclusion, each participating patient, GP, and staff member at the nursing home signed an informed consent form.

### Descriptions of study participants

From February 2010 through April 2011, nine patients and their respective GPs were included, consecutively, in the study. The mean age of the patients was 64 years (44–94 years), and that of the doctors was 51 years (34–61 years). The mean duration of the doctor–patient relationships was 15 years (3–25 years). The patients admitted had differing primary diagnoses, except for two, whose main diagnosis was multiple sclerosis (Table I). For all patients, the central aim of their stay was rehabilitation. For patient B, D, and H, the aim was also to provide needed relief to their usual caretakers.

**Table I:** Characteristics of the participants

	Patients					General practitioners			
			
Participants	*Gender*	*Age (years)*	*Civil status*	*Medical diagnoses*	D/P relationship (years)	*Gender*	*Age (years)*	*Listed patients*	*Doctors at the office*
A	M	83	Married	Parkinsons disease	25	M	58	1850	3
B	M	44	Married	CFS	23	M	61	1100	5
C	M	58	Single divorced	MS	13	M	51	1550	4
D	M	58	Married	Stroke	10	M	53	1300	7
E	F	46	Single divorced	Sequels; brain abscess	18	F	53	1300	4
F	M	84	Married	Hip fracture	24	M	57	1400	5
G	M	57	Single	Chronic pain, abuse	10	F	49	1500	5
H	F	52	Married	MS	11	M	42	1850	4
I	F	94	Widow	Glaucoma, advanced age	3	F	34	1650	5

CFS=chronic fatigue syndrome, MS=multiple sclerosis, D/P-relationship=duration of doctor–patient relationship, M=male, F=female.

## Analysis

The analysis was performed by the first and second author who included the other authors in consecutive discussions for clarifying and refining the issues in question. All the authors have extensive clinical experiences as GPs and doctors in primary care, and three of them are also experienced researchers and academic teachers. The first author has worked in the double position as a regular GP and a part-time consultant physician in a nursing home for longer periods. Her repeated observation of a certain informational “gap” between primary care and institutional care had engendered the current project (Mjølstad et al., 2013a).

The first steps of our analysis of the GP and patient interviews, inspired by a hermeneutical canon developed by Kvale (1983, 1996), have been presented in a previous paper dealing with the difference between GPs’ believed and actual knowledge about their patients (Mjølstad et al., 2013b). The first analytical level dealt with the participants’ self-understanding while the second level was based on critical common sense understanding (i.e., critical understanding of what is being said by using general knowledge/common sense). This approach was double-layered, guided by the questions “what does the person state about the matter at hand?” (objective approach) and “what does this statement say about the person?” (subjective approach). Finally, in the third analytical level, we aimed at understanding these findings through the application of existing theories.

In the current paper, based on the previous analyses of two texts (telephone interview of the doctors and first interview of the patients), and supplied with three other texts (excerpts from the medical records, the biographical records and second interview of the patients), we performed a comparison of what we, according to Mishler (1986), refer to as three different “voices.” For this purpose, the interviews were compared topic by topic with regard to concurrence or divergence between the GP and the patient as to the most essential elements of the rehabilitation (for description of the analysis step by step—see Figure 1. Further details have been elaborated in Appendix). Any lack of salient information and/or attention to specific, significant details which the GP exhibited was also identified. Both the GPs’ and patients’ concurring and diverging statements were compared to the institution's interpretations of the information provided, as reflected in the institutional rehabilitation plans. These plans included certain explicitly stated, standard forms of intervention. Other treatments and interventions that were less explicitly offered, was deduced from the first author's field notes and from the patients’ medical records. This part of the analysis involved de-contextualizing and re-contextualizing both the observed and the recorded elements, examining both the structural and the habitual aims as they manifested in the routines. Finally, we integrated these findings into theoretical frameworks, exploring the balance between the three voices. Here, we applied the distinction Elliot G. Mishler (1984, 1986) introduces regarding the patient's voice as the voice of the lifeworld, a first-person account, with the institution's voice as the voice of medicine, a third-person account. The GP acquires an “in-between” position: partly third-person—the professional voice of medicine—and partly first-person—the personal voice of someone acquainted with the patient's lifeworld.

## Results

We now present and compare, in condensed form, the three different elicited perspectives on the participating patients’ needs and aims upon their admission to the rehabilitation unit: *the* *GPs’ recommendation*s, *the patients’ own wishes and the institution's priorities*, *and the therapies actually chosen for and implemented in the rehabilitation plans*. Subsequently, we focus on certain specific patient wishes documented in the biographical record and presented to the staff by the researcher. We examine these in terms of the relevance such wishes hold for the overall aims of the rehabilitation process, and the degree to which they are consistent with what a typical, contemporary, rehabilitation institution might be expected to offer, in terms of capacity and mandate.

### The GPs’ recommendations

The GPs formulated an “optimal rehabilitation plan” for specific patients based on their personal knowledge, detailing their specific needs while also taking into account the patients’ personality and life circumstances. However, as revealed in a comparative analysis of the GPs’ recommendations versus the patients’ wishes, the degree to which the GPs were capable of recommending measures that coincided or harmonized with their patients’ own wishes differed markedly. Those GPs who had developed a personal, long-term doctor–patient relationship were able to formulate recommendations that harmonized better with the patients’ own preferences than did those of GPs who were less familiar with their patients’ lives. In those cases in which the clinical relationship was less developed (although it could have been long-lasting), the GPs tended to recommend non-specific measures, seemingly based on professional assumptions regarding the types of services a rehabilitation unit might be expected to offer routinely. Further details concerning the participating GPs’ actual knowledge of their patients as persons have been published elsewhere (Mjølstad et al., 2013b).

### The patients’ expressed wishes

Given sufficient time and opportunity to elaborate on their reflections, and despite certain physical and/or mental impairments, all of the patients proved able to express detailed, comprehensive and coherent descriptions of their specific needs for the rehabilitation stay. Subsequently, they were willing to have this information passed on to the staff in the form of biographical records. Certain of the patients’ wishes could be incorporated easily into the standard institutional program by making relatively minor adjustments. For example, one patient requested receiving physiotherapy later in the day to avoid getting up early in the morning. Other patients requested that the staff familiarize themselves with details regarding their daily routines. A wide variety of issues proved to lie at the core of the patients’ actual needs; the specificity of these could be seen as mirroring fundamental, preexisting realities within their lifeworld. Some of these will be elaborated below.

### Interventions actually implemented by the institution

**Figure 2:**
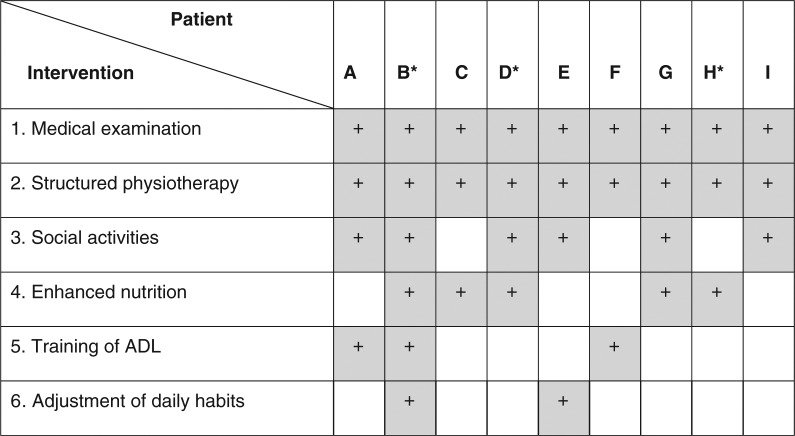
An overview showing what kind of interventions (1–6) the patients (A–I) received at the nursing home during their stay. Grey [+], intervention determined; white [], intervention not established; [*], the rehabilitation admissions of patients B, D, and H were motivated in part by the needs of their primary, daily caretakers for relief.

In accordance with the rehabilitation unit's daily routines, the nurses encouraged all patients to participate in common meals and social activities, as well as to be physically active generally. In addition, they systematically observed and recorded in detail how much time the patients spent in their rooms, the group activities they attended, whether they ate and drank sufficiently, and the extent to which they communicated with fellow patients and received visitors. When determining the individual patients’ rehabilitation plans, the staff drew from a limited number of standard interventions (Figure 2). Upon admittance, all patients underwent a thorough *medical examination*, performed by the unit's consulting physician. The staff all agreed as to the relevance for all patients of *structured physiotherapy*, and all patients received input from the unit's physiotherapists at some point during their stay. Most patients, particularly those considered to be at risk of suffering from “loneliness,” were explicitly encouraged to participate in *social activities* (common meals, group gymnastics to music, entertainment, etc.). Certain patients were singled out to receive special care: (1) *enhanced* *nutrition*—increasing their food consumption, and/or supplementing their diets with nutrient-rich food or drinks, and/or modifying their diets, for example, in cases of diabetes; (2) *training of ADL*—including dressing, eating, and personal hygiene; and/or (3) *adjusting daily habits*, such as receiving help to rise earlier and/or observe more regular sleep habits.

### Standardization and stories

The in-depth interviews with the patients, the first-person accounts, proved at times to be the only source of knowledge about very specific personal needs, information that was not mentioned by their GPs, and neither identified nor addressed by the institution. These related equally to two types of patient requests: those within the scope of what the standardized institutional treatment repertoire was equipped to identify and respond to, and, those raising issues which warranted a frame of understanding and a repertoire of responses which might be seen to extend beyond the purview of this type of institution.

### Patient wishes falling within the scope of the institution's customary repertoire

When examining how a standardized repertoire of interventions was implemented at the individual level, we looked at three categories—physiotherapy, social activities, and nutrition—and found what we have termed an implicitly double-layered standardized repertoire. That is, not only was the division of intervention categories as such standardized, but the approaches within each category were also standardized, despite the obvious feasibility of individualized adjustments being made. This can be seen in the following examples reflecting the institution's responses to the wishes patients had expressed in their first-person accounts.

#### Personal aims regarding physiotherapy

The staff's emphasis on structured physical training supervised by a physiotherapist seemed to suit the initial wishes of most patients. However, it soon became clear that they also had preferences as to *how* they were to be trained and assisted by the physiotherapist. All patients had articulated various aims for their physical rehabilitation, described in the biographical records. Despite the staff being explicitly trained and educated to formulate plans adapted to individual patient's needs, and despite procedural documents encouraging them to do so, the patients’ expressed preferences were almost never acted upon by the staff.

Patient F was a recently operated, 84-year-old man who, when interviewed, elaborated detailed preferences for his rehabilitation stay to include solutions that had been worked out for him at home. There, a special walking aid made it possible for him to go out into his yard and around his house by himself, allowing him to enjoy the garden and a terrace which his son had recently constructed for him. This practical and relationally meaningful physical aid was not integrated into his individualized treatment plan, despite having been documented in his biographical record.

Patient H was a 52-year-old woman suffering from severe MS who was eager to exercise using a stationary bicycle. Her explicit goal of counteracting her restricted mobility was jeopardized by a technical mismatch between her wheelchair and the exercise bicycle's pedals. The physiotherapist did not prioritize solving this problem but rather focused on the patient's spastic paralysis, which was deemed more urgent to treat. Consequently, patient H was the passive recipient of stretching (massage) yet was hindered in taking the initiative to exercise actively by herself—despite the importance the unit claimed to ascribe to such independent activities.

#### Patient wishes in relation to social activity

The unit staff actively encouraged the patients to take part in common meals and social activities as well as to communicate with one another. Although clearly focused on observing and documenting the social behavior of each patient, the staff did not seem to consider what each individual patient might deem to be meaningful activities. Nor did they take into account variations in the patients’ ages, personality, or interests, which, in all cases, had been detailed in the biographical records.

Though patient D, a 58 year-old man, described himself as a social person, he was very determined to decide for himself with whom to interact. He refused to allow the staff to couple him randomly with patients he didn't know, stating that he was fully capable of establishing contact on his own, but only if and when he were to encounter someone he considered interesting to talk to.

Patient E was a 46-year-old woman who, during the first interview, had shared her fears that her increasing incapacitation would cause her to become ever more isolated. She very much wished for help to go to a cinema and to find other ways to socialize with people her own age. That her innately social nature and need for physical training were so compatible with the unit's standardized programs, might have contributed to her specific personal ambitions and wishes not being taken into consideration.

#### Personal needs regarding nutrition and diet

Nutrition was another central topic for the rehabilitation unit, as patients might arrive either underweight or obese, though for very different (underlying) reasons. Consequently, any potential improvement would require nutritional approaches that were customized and contextually meaningful.

Patient G was a 57-year-old man who suffered from intractable chronic pain. He was also seriously underweight, which presented an obstacle to his undergoing a surgical intervention which could potentially reduce his pain. He usually gained weight during his stays at an institution because, he said, his appetite and well-being improved greatly when he was feeling less lonely than he did at home. Nonetheless, the unit did not—or could not, due to standardized restrictions in the length of admissions—offer to extend his stay in order to help achieve a sustainable improvement in his general state of health.

Patient D had had a stroke seven years earlier, forcing him to use a wheelchair. Since then, his weight had increased and he very much wanted to be put on a diet. He feared that he would literary “grow out of” his wheelchair; using a larger one would require him to widen all the doorways in his house. This was an expensive procedure, and one which he had already had to go through after the stroke. Despite this explicit wish, no tailor-made, long-term weight reduction plan was established for him during his stay.

### Patient wishes extending beyond the scope of the institution's customary repertoire

Some of the patients’ wishes and requests might be seen as extending beyond the scope of the standardize repertoire of this type of rehabilitation institution. Such needs involved highly specific concerns and existential issues (complexes of values and meanings), the subtlety of which only became apparent when the researcher had access to relatively detailed information regarding the patients’ particular lifeworlds. Some information of this sort was provided to the researcher by the patients themselves during the interviews. Some of it emerged during the short telephone interviews with the patients’ GPs, in cases where a well-established doctor–patient relationship existed. The GPs in cases A, B, C, for example, had all known their patients for a long time, and there was clear doctor–patient agreement as to what was at stake. Though some of the patients’ wishes were far from concrete, they could nevertheless have been attended to, given a flexible mind-set and time to discuss them with the patients. The following stories illustrate such complex constellations.

#### Fear of being abandoned

Patient A, an 83-year-old man suffering from Parkinson's disease, was in need of rehabilitation. He usually lived at home with his wife, his main caretaker. The patient's need for comprehensive and reliable care was considerable. GP A perceived that the high level of strain in his marital relationship was a topic which would be crucial for the health personnel at the rehabilitation unit to bring up and respond to since it posed a threat, potentially jeopardizing not only the man's confidence but also his actual safety. When asked by the researcher about his situation at home, patient A quite frankly confirmed the GP's concerns and his own fear of being abandoned as follows: *To be honest, I'm afraid our relationship is over—there'll be a break-up. I feel desperate!* Referring to fruitless attempts to enter into a dialogue with his wife on this matter, he stated: *My wife is quite an introvert. I don't manage to get close enough to her to talk about this*. In addition to his fear of being abandoned by his wife, he also expressed a worry that death from Parkinson's, his main diagnosis, was imminent. Although these existential matters were clearly documented in the biographical record, and brought up explicitly by the researcher during meetings as being important human concerns, the topics were never addressed by the consulting physician during the patient's stay. One reason the doctor gave was that it would have been too time-consuming. Also, such issues might be regarded as falling within the purview of the patient's GP; consequently, the biographical record was included in the discharge report the institution provided to GP A.

#### The importance of being trusted and believed

Patient B, a 44-year-old man, lived at home with his wife and two children. Chronic fatigue had dramatically impaired his capacity to function, forcing him to stay in bed most of the time and causing him to have to struggle to coordinate his daily rhythm with his family's everyday activities. The fact that examinations at several hospitals had failed to yield any unambiguous diagnostic results provoked scepticism among medical staff regarding the nature of the patient's problems. GP B stated: *Patient B is very concerned about being believed because he has previously experienced the opposite*. GP B was concerned that the patient would equate his sense of being judged for not “really” having a disease with not being taken seriously as a human being. Consequently, GP B considered it crucial to any successful rehabilitation that the patient be perceived and treated by the staff as reliable and trustworthy. The importance of being believed was explicitly confirmed by patient B in the interview: *The last time I was here, one doctor actually came to my room and told me that some of the staff doubted that there was any valid medical explanation for my symptoms or disease*. In addition to the patient's fundamental need to be met as “a person with credibility” being documented in the biographical record, existential worries about the future were also revealed. Much to the patient's surprise, these worries were interpreted by the consulting physician, with no further exploration of the patient's lifeworld, as being “depressive thoughts.” A personal, *meaning-laden*, existential worry was thus translated into a generalized and depersonalized medical category: depression. Had the staff invested more time in talking to him, they might more likely have interpreted his concerns as existential rather than as indicating a depression. During his stay, patient B's wish not to be confronted with doubts surrounding his disabling condition was never addressed explicitly. The institution may have responded implicitly, however, given that he reported no incidences of remarks or offending discussions as having taken place during the present stay.

#### A wish to be “pushed” but in a tailor-made and respectful way

Patient C, a divorced 58-year-old man with MS, usually capable of taking care of himself, was now in the need for rehabilitation. Patient C had known his GP for 13 years, and had shared very personal problems with his doctor. GP C emphasized that the disease had “transformed” the patient from being strong, sociable and independent into being weak, dependent, and self-pitying. GP C stated: *I've tried to focus on his strengths and be supportive. And I've told him to stop feeling sorry for himself!* When his GP's reflections were shared with patient C, he confirmed and also commented on the GP's strategies to motivate him: *GP C was right of course—to tell me to stop feeling sorry for myself. And he got me going again. But he couldn't have said that if he hadn't known me so well*. GP C deemed it important for patient C to be supported in interests and activities that he found pleasurable. Although the patient basically agreed, he stated explicitly that such a resource-oriented approach would only work if he were “pushed” into tailor-made activities—in a non-patronizing and trusting manner, which could, however, be both frank and firm. Under those circumstances, he believed, he would be able to avoid succumbing to depressive moods, passivity, or hopelessness. The institution did not seem to have much to offer in response to this wish. The patient complained of being “bored stiff” during his stay and was so dissatisfied that, at one point, he wanted to leave the unit. The solution found was to grant him several “leaves of absence” to go home, watch the soccer matches he was interested in, be with his friends. The result was that he was more often absent than present, which interfered with the routines at the unit and frustrated the staff.

### Observable mismatches between stories and routines

To sum up the results, a series of minor and major mismatches could be observed between the GPs’ recommendations and patients’ wishes on the one hand, and the institution's actual rehabilitation treatment schema on the other. Although the rehabilitation unit's procedural documents formally commit the institution to delivering *individualized* care, it was evident that those treatment interventions which were actually implemented were, in reality, individualized to only a very limited degree. This was so even in situations where the expressed wishes of the patients regarded one of the core institutional activities, such as physiotherapy, nutrition, and social engagement. The detailed content included within each of the standardized categories of intervention remained relatively fixed as well, despite the obvious feasibility of individual adjustments being made. The researcher was typically told that the biographical document was valuable and relevant; this was said also in situations where it had highlighted patient wishes and needs of a more personal, even existential, nature, which would thus have demanded an even more highly individualized flexibility and engagement on the part of the staff. Nonetheless, the institutional responsiveness was limited, as can be deduced both from the records and from the patients’ final reports during the second interviews.

## Discussion

Our study indicates that the premises for rehabilitation, “a process of enabling someone to live well with an impairment in the context of his or her environment and, as such, requires a complex, individually tailored approach” (Hammell, 2006) might not be adequately met, even when individualized care is a stated goal. This ambition proved to be more of a professional vision than an actual clinical reality. Our findings raise a variety of questions. We have chosen to reflect on three: (1) What lies at the core of the institution's reluctance or inability to implement genuinely individualized care? (2) Are there arguments to support relational and existential issues being addressed in a rehabilitation institution? and (3) If this were to be recommended, might it also be wise, structurally, to train the patients’ regular GPs to serve as consultants to the process of eliciting details (with patient consent) of the individual patient's needs and resources? We'll use an excerpt from the material regarding one of the nine cases to open our exploration of these three questions (see Box 1).

**Box 1. An illustrative scene**

Patient A's biographical record, describing his strained marital relationship and his existential fear that death from Parkinson's disease was imminent, was presented to the staff in a meeting. Even though these issues were overtly acknowledged as being of significant human concern, they were never addressed during the patient's stay. This is confirmed in the following dialogue between the researcher (I) and patient A (PA):

*I: Did the consulting physician talk to you about these matters?*

*PA: Well – hello! [Ironic, meaning “No way!”]*

*I: So the doctor didn't talk to you?*

*PA: The doctor came by my room the other day and asked; “How are you doing?” What else could I answer but: “Fine - under the circumstances.”*

*I: So you did have a conversation with the doctor?*

*PA: I wouldn't call it a conversation. The doctor just popped in and then left*.

### Why was genuinely individualized care not implemented?

#### A staff perception that the treatment was, in fact, individualized

In dialogues with the researcher, the staff typically emphasized lack of *time* as the main obstacle. We presume, however, that more complex barriers might be involved. To begin with, the staff might have perceived the institution's treatment plans to be relatively customized since all patients had routinely been given a questionnaire about their personal aims for their stay. Furthermore, the staff might have interpreted the fact of the patients receiving differing sets of activities from the institution's standardized repertoire as indicating that their treatment had been individualized.

#### A disease-oriented, biomedical focus on cure

We suggest that, at its core, the lack of concrete responses to patients’ expressed wishes and needs might reflect the dominant, disease-oriented mindset associated with scientific biomedicine as it relates to the concept of *cure* (Barbour, 1995; Baron, 1992; Cassell, 2004; Montgomery, 2006; Toombs, 2001; Zaner, 2003b). Several scholars have conceptualized biomedical and humanistic therapeutic approaches, associated with *cure* and *care* respectively, as being complementary within Western health care systems (Miles & Mezzich, 2011; Silva, Charon, & Wyer, 2011). The therapeutic, that it is, cure, concept has the objectified, material, physical body as its scientific basis (Leder, 1992); evidence-based interventions, from so-called evidence-based medicine (EBM), have become the gold standard within this realm. The concept of care, on the other hand, is based on methods for appraising subjectivity, including relational and social phenomena (Montgomery, 2006). To reconcile these differing views, a patient-centered model (Levenstein et al., 1986) has been conceptualized, suggesting that two parallel “agendas” (the doctor's and the patient's) should be allowed to evolve and eventually fuse during the medical encounter (Miles & Mezzich, 2011). “Patient preferences and values” are also emphasized in models of evidence-based practice (“The EBM flower”) (Haynes, Devereaux, & Guyatt, 2002). However, the fundamental clinical validity of the hegemonic epistemology of biomedicine as such (the basis for EBM) has rarely been challenged (Kirkengen et al., 2013). Consequently, the discourse on “patient preferences and values,” and the associated training in patient-centered communications, typically aims at eliciting patients’ views and preferences with reference to biomedically defined problems and options. Very little emphasis has hitherto been put on teaching and training doctors to recognize and address more fundamental existential issues as they pertain to a patient's subjective life-world. The medical relevance of such issues is, however, becoming consistently more evident, as we will later explain. In the Norwegian context, health care researcher Marte Feiring (2012) has asked if it is possible to increase governmental control and oversight while simultaneously enhancing user involvement and empowerment. It is certainly difficult to be guided both by group-based, scientific evidence and by the subjective opinions of the individual user. If these principles, which are cornerstones of rehabilitation in Norway, appear contradictory or even incompatible, which of them should be given precedence? Or, from a different perspective, what is needed to unite seemingly incompatible principles in order to prevent the fundamental aims of the overall effort from being jeopardized?

#### Epistemological obstacles to actual patient involvement and “empowerment”

The term “to empower” is ambiguous, implying both that power is at stake and that someone “in power” may be willing to renounce it (or some of it) on behalf of someone less powerful or even powerless. Implicit in the notion of “empowering patients” is the fact that medicine does hold power, a reality that has been broadly discussed within sociology, anthropology, and philosophy (Zaner, 2003a). The main source of this power has been identified as being the type of knowledge about the human body which medicine is mandated to administrate, and the type of knowledge production, grounded in scientific methodology, which it applies (Foucault, 1975). Medical professionals certainly recognize an asymmetry in the amount of knowledge doctors and patients have. However, the fact that their professional knowledge, grounded in the sciences, is presumed to be value neutral seems to help them remain unaware of the power inherent in the objectifying biomedical episteme as such (Foucault, 1975; Faubion, 2000). Other scholars have explored the impact of the biomedically framed and asymmetrical doctor–patient relationship with regard to certain non-objectifiable phenomena in human illness (Frank, A.W., 1991, 2007; Kleinman, 1988; Toombs, 1992). Correspondingly, philosopher Pierre Bourdieu has explored what he calls “habitus,” in the sense of particular habits resulting from professional training and socialization; these manifest as incorporated “ways of doing” that are no longer reflected upon but simply presumed to be correct and adequate (Bourdieu, 1990). Such “habits” might be expressions both of explicitly assigned power (the right to decide) and of implicit power, that is, the authority to define the nature of a problem and determine what should count as relevant. Such convoluted power is elucidated by Norwegian physician and philosopher Kari Agledahl, who, based on observations of doctor–patient consultations, demonstrated a habitus of *polite avoidance* when it came to engaging in patients’ existential concerns (Agledahl, Gulbrandsen, Forde, & Wifstad, 2011).

### Are there arguments to support relational and existential issues being addressed in a rehabilitation institution?

#### Support from science

Until fairly recently, there was only a small body of medically authoritative, biologically based evidence to support the claim that lifeworld phenomena matter to overall, clinical outcome, including in a literal, biological sense. During the last decades, however, empirical knowledge has been accumulating, showing that—and in increasing detail also how—a person's lifeworld experiences have direct impact on that individual's body, down to the sub-cellular level (Getz, Kirkengen, & Ulvestad, 2011; Tomasdottir et al., 2013). It has now been demonstrated beyond doubt that relational and social matters are of general medical relevance (Blackburn & Epel, 2012; Danese et al., 2009; Friedman, Karlamangla, Almeida, & Seeman, 2012; Gruenewald et al., 2012; Kiecolt-Glaser, Gouin, & Hantsoo, 2010; Surtees et al., 2011). This long-avoided topic within medical knowledge production is fast becoming obligatory, seen now as an essential component of adequate medical comprehension. Such knowledge may also be of particular relevance to the care of frail and decompensated persons (Clegg, Young, Iliffe, Rikkert, & Rockwood, 2013; Gruenewald, Seeman, Karlamangla, & Sarkisian, 2009; Kuchel, 2009; Szanton, Allen, Seplaki, Bandeen-Roche, & Fried, 2009). Given the mounting evidence of close links between existential strain and ill health, we assert that all medical institutions should be prepared to consider the health implications that hardships and other life experiences have on the persons in their care. This is particularly relevant for institutions specially “designed” to rehabilitate frail and decompensated people, to assist them to recover and maintain the spectrum of capacities and functions required for them to return to their homes and enjoy their privacy and independence as long as possible. It is our contention, consequently, in response to the second question engendered by our study, that research does support that such issues should be addressed. The question is how and, perhaps, by whom. Implicit here is the contention that standardized programs for such patient groups are highly inappropriate. Person-specific and context-specific measures must be applied if the medical intervention of “rehabilitation” is to be successful and sustainable. Western societies, despite limited resources, have to care for a growing patient group characterized by advancing age, complex morbidity and the desire to enjoy living independently as long as possible. To face these challenges, new modes of collaboration within health care systems are now being developed. Standardized interventions and routines may seem to be a feasible, cost-effective and reasonable way to meet the demands of care and transition. However, adherence to such standardized interventions and routines might prove inadequate to meet the diversity of specific needs that characterize that patient group (Rosstad, Garasen, Steinsbekk, Sletvold, & Grimsmo, 2013). According to the late Norwegian scholar Harald Grimen (2009): *Routines are double-edged swords. They facilitate work but restrict the field of vision. Routines can bring both mental comfort and medical (and juridical) disaster. This is the paradox of routinization: What makes routines helpful also makes them dangerous*.

#### Support from human rights

Another argument for professionals to prepare to address existential issues in settings such as a rehabilitation unit, and in care for the elderly in general, is found in recent Norwegian legislation. Here, the explicit political emphasis that is placed on the relationship between dignity and existential questions coincides with the increasing focus within medicine on the relationship between health and experiences. A governmental document entitled “Verdighetsgarantien” (“The Right to Dignity”) (Helse og Omsorgsdepartementet, 2010) acknowledges elderly people's rights to privacy and autonomy, to participate actively in individualized service or care, and to receive qualitatively appropriate assistance. The explicitly stated intention is to “safeguard security and ensure the possibility for each individual to lead a meaningful life.” An explicit institutional obligation *to facilitate and participate in dialogues regarding existential matters* (§ 3) is also affirmed.

### A future role for GPs as “negotiators of personal knowledge” during transit situations?

In one of this project's previous sub-studies, a group of seasoned, urban GPs expressed a high level of engagement with and interest in their frail and/or elderly patients. They stated that they would be more than willing to make “strategic” consulting visits whenever their most vulnerable patients were admitted to a rehabilitation institution or nursing home (Mjølstad et al., 2013a). The GPs perceived this to be a more cost-effective use of their professional time than participating in the many compulsory “co-operation meeting activities” currently mandated by the Norwegian health and social care system. The present study adds depth to that perception. We were able to show that, even in the absence of specialized, formal training, and even in the context of only a brief telephone interview, experienced GPs were able to contribute important information about their patients as persons, knowledge which clearly extended beyond information that is customarily considered “medically relevant” for transmittal between actors in the health care system. Any new, professional routine wherein GPs would be expected to contribute “personal” information about their patients would obviously require patient consent. It would also presume that the doctor had received adequate education and training. In our opinion, the present study gives reason to believe that GPs might thus become valuable advisors in the process of discerning which issues in human biographies are most salient with respect to health. Such issues might be particularly useful to shed light on situations in which a person's health has decompensated for reasons that are difficult to identify when viewed from a traditional biomedical perspective.

In the debate (Miles & Mezzich, 2011) that has been going on since George L. Engel proposed “the bio-psycho-social model” as an appropriate framework for medical encounters (Engel, 1977), various scholars have pointed to limitations in the model as such, in particular, its “lacking dimensions” regarding the existential and spiritual realms of human life. One predictable consequence of these debates has been the “appending” of the word “spiritual” to the model's “bio-psycho-socio” title (McCullough, Hoyt, Larson, Koenig, & Thoresen, 2000; Powell, Shahabi, & Thoresen, 2003; Sulmasy, 2002). The unresolved epistemological shortcomings of the original concept, however, have hardly been addressed. To simply add a human dimension that is conceptualized, philosophically, as separate, does not address or account for the experiential unity of being-in-the-world as “Me,” which endows every human being with a unique “core sense of mineness,” as ethicist Richard Zaner (2003b) has termed it. It is precisely this corporeal being, this “mineness” of the human body that has been shown to be of central medical relevance.

Recent efforts to improve the way medicine meets the challenge of the suffering human being are giving rise to various “movements” which might ultimately contribute to radical changes in the medical encounter as well as profound enrichment of the therapeutic repertoire. One of these movements, “person-centered medicine,” aims at making doctors more aware that implicit in each medical encounter is the presence of two *persons*: the patient and, on an equal level, the physician. Another movement, “narrative medicine,” aims at giving the diseased person, the suffering subject, the possibility to make sense of her/his situation, to tell and to be heard (Frank, A.W., 1998). In addition to acknowledging the subject's right to voice her/his own experience, the listening professionals must also deepen and refine their empathic abilities if they are to understand what they hear. Narrative competence, that is, the empathic ability to recognize relevant patterns in other human beings’ life stories, can both be learned and taught (Charon, 2012). At the same time, it is of paramount importance neither to reduce empathy to merely another instrumental skill (Macnaughton, 2009), nor to confuse it with sympathy or identification. Empathy, as understood within the phenomenological tradition, particularly as elaborated by scholar Edith Stein, means to appraise another person's “otherness” (Frank, G., 1985). This crucial “open-mindedness” on the part of the medical professional is echoed in Richard Baron's (1985) seminal paper entitled, “I can't hear you while I'm listening”. French philosopher and psychiatrist Pierre Janet (van der Kolk & van der Hart, 1989) has traced the detrimental impact it has on health for people to be prevented from telling and being listened to as they attempt to come to grips their own experiences, especially those which engender existential upheaval. The work of psychologist James Pennebaker (2000), among others, has demonstrated the health benefits of *formulating a narrative*, including its impact on reducing stress and physiological overtaxing.

We may now conclude that, in order to provide effective and sustainable health care, current general practice as well as institutional norms should be expanded to encompass “personal” topics, in the sense of their being relational and existential. The question will arise, of course, as to how to identify those patients who are most likely to benefit from this kind of attention and help. Our study has shown that a simple “screening” approach is unlikely to yield that desired clarity; we observed the lack of effectiveness both of routinely questioning patients about their own “aims” for their stay at the institution and of the consulting physician's informally visiting the patient's room as part of a busy schedule (Box 1 at start of Discussion). Both the patient's capacity to conceptualize and express those existential phenomena which have clinical significance, and the health care worker's capacity to identify them, are likely to be enhanced considerably through the investment of time, and with increased interpersonal experience and trust. Here is where we envisage a potential future role for GPs, when their primary focus on diseases themselves shifts to emphasize knowledge of the individual *persons* who suffer from these diseases (Starfield, 2011).

## Methodological considerations

The strength of our study lies in the way its design, method, and analytical framework enabled us to capture differing perspectives on the needs of frail individuals at a rehabilitation institution. Even though the participants were consecutively included in the study (as opposed to strategically), we obtained a varied sample of informants, representing a diversity of experiential backgrounds; this also helped counterbalance the low number of participants. The study yielded insight into how the wishes and needs of the patients were informed by the specifics of their lifetime experience and their everyday lifeworld, and provided nuanced knowledge about the complexity of the rehabilitation process. As to the transferability of these results to other similar groups, the individual situations of study participants and the routines at institutions will, obviously, differ. Based on our clinical experience, however, neither the range of patients nor the nature of the institution stands out as being unusual. The limited time available for telephone interviews with the GPs (10–15 min) might have impacted their capacity to articulate recommendations for their patients. On the other hand, this might bode well for the prospects for transferability of the results since such stringent time constraints exist in real life practice. It is also possible that even better results might be seen in the future using this same time frame if routines were formally established and acknowledged so that the GPs expected, and therefore were mentally prepared, to take a role as “consultant” for patients in transit, as described in this paper. The detailed and comprehensive field notes contributed valuable insights into the institution's routines and the medical records. More consistent observation of the interactions among staff members, and/or additional interviews with them, might, however, have yielded deeper or more differentiated insights into the rationale informing their actions. In accordance with the traditions of phenomenological–hermeneutical research, we have made our position explicit and have aimed for methodological transparency. We have integrated our findings using relevant theoretical frameworks to unfold their implicit features, well aware that our conclusions are tentative and represent only a selection of a wider range of possible interpretations.

## Conclusion and implications

In the present study from a rehabilitation unit, we found that the institutional voice of medicine consistently tends to override the voice of the lifeworld; that is, patients’ stories became subordinate to the institution's routines. Consequently, despite the best of intentions and the application of the best knowledge according to current standards, the overall aim of health care seeking to provide appropriate rehabilitation to frail and decompensated patients in order to help them return to their everyday life at home might have become jeopardized to some extent. We suggest, therefore, that a “closer look *and* a wider view” might be well worth trying in the future. By this we mean: (1) a closer collaboration between the GP and the institution to elicit and explore information as to the specific context of each individual patient, and (2) a more flexible and openly *person*-oriented (in addition to the more limited and standardized *patient*-oriented) conceptualization and application of patient treatment care plans so that they are more genuinely tailor-made to better represent the “best possible effort/approach to suit this specific person's lifeworld.” When health personnel do not know about their patients’ life circumstances, mere chance will determine whether the treatment measures selected are the optimal ones. Or rather, the probability of knowingly selecting optimal, or even adequate, treatment measures will be low.

## Appendix

Example of stepwise analysis patient A.

**GP A's recommendations:**

*-Important to focus on the stressful home situation involving marital strain*.

*-Important to provide relief for caretaker (wife)*.

**Patient A's expressed concerns and wishes:**

*-Worried about the difficult situation at home due to marital strain*.

*-Existential worries regarding sickness and death due to Parkinson's disease*.

*-Desire to receive physical training to improve his ability to walk*.

**Patient A's biographical record:**

“Patient A is worried about his strained marriage and very difficult home situation. He wants to receive physical training to help improve his ability to walk. He has many questions about his chronic disease; he knows two people who died from Parkinson's and is anxious regarding whether he too will die of the disease. His GP emphasizes that the most import issue to address during the patient's stay is how to safeguard his care in the future, which seems endangered by marital strain.”

**Actual interventions as identified in patient's medical records:**

*-Medical examination (report from consulting physician)*

*-Structured physiotherapy (report from physiotherapist)*

*-Social activity, training of activities of daily living (ADL) (reports from nurses)*

**Observation concerning the actual interventions as recorded in the field notes:**

“The consulting physician has not talked to the patient about his stated concerns and neither has anyone else (nurses).”

**Patient A's comment on actual interventions (from vignette in** Box 1 **):**

*I: Did the consulting physician talk to you about these matters?*

*PA: Well – hello! [Ironic, meaning “No way!”]*

*I: So the doctor didn't talk to you?*

*PA: The doctor came by my room the other day and asked; “How are you doing?” What else could I answer but: “Fine – under the circumstances.”*

*I: So you did have a conversation with the doctor?*

*PA: I wouldn't call it a conversation. The doctor just popped in and then left*.
